# Immunocompromised Children with Severe Adenoviral Respiratory Infection

**DOI:** 10.1155/2016/9458230

**Published:** 2016-05-08

**Authors:** Joanna C. Tylka, Michael C. McCrory, Shira J. Gertz, Jason W. Custer, Michael C. Spaeder

**Affiliations:** ^1^Department of Pediatrics, Section of Pediatric Critical Care, Rush University Medical Center, Chicago, Il 60612, USA; ^2^Department of Anesthesiology, Section of Pediatric Critical Care, Wake Forest School of Medicine, Winston-Salem, NC 27157, USA; ^3^Department of Pediatrics, Hackensack University Medical Center, Hackensack, NJ 07601, USA; ^4^Division of Pediatric Critical Care Medicine, University of Maryland Medical Center, Baltimore, MD 20201, USA; ^5^Division of Pediatric Critical Care, University of Virginia Children's Hospital, Charlottesville, VA 22908, USA

## Abstract

*Purpose*. To investigate the impact of severe respiratory adenoviral infection on morbidity and case fatality in immunocompromised children.* Methods*. Combined retrospective-prospective cohort study of patients admitted to the intensive care unit (ICU) in four children's hospitals with severe adenoviral respiratory infection and an immunocompromised state between August 2009 and October 2013. We performed a secondary case control analysis, matching our cohort 1 : 1 by age and severity of illness score with immunocompetent patients also with severe respiratory adenoviral infection.* Results*. Nineteen immunocompromised patients were included in our analysis. Eleven patients (58%) did not survive to hospital discharge. Case fatality was associated with cause of immunocompromised state (*p* = 0.015), multiple organ dysfunction syndrome (*p* = 0.001), requirement of renal replacement therapy (*p* = 0.01), ICU admission severity of illness score (*p* = 0.011), and treatment with cidofovir (*p* = 0.005). Immunocompromised patients were more likely than matched controls to have multiple organ dysfunction syndrome (*p* = 0.01), require renal replacement therapy (*p* = 0.02), and not survive to hospital discharge (*p* = 0.004). One year after infection, 43% of immunocompromised survivors required chronic mechanical ventilator support.* Conclusions*. There is substantial case fatality as well as short- and long-term morbidity associated with severe adenoviral respiratory infection in immunocompromised children.

## 1. Introduction

Adenoviruses are a group of DNA viruses that are estimated to cause 5–10% of febrile illnesses in infants and children [1]. Infections occur primarily in children less than five years of age and may involve the respiratory tract, conjunctiva, or gastrointestinal tract or become disseminated. Adenovirus is a common cause of acute respiratory infection manifesting as bronchiolitis, wheezing, croup, or pneumonia [2–5]. In immunocompetent hosts most infections are self-limited; however respiratory infection from adenovirus can be severe requiring pediatric intensive care unit admission [2], mechanical ventilation [2, 3, 6], and extracorporeal life support [7].

Children are at risk for long-term respiratory sequelae from adenoviral respiratory infection. Adenoviral infections have been linked to the development of chronic pneumonia, bronchiectasis, and hyperlucent lung [8–11]. Adenovirus is the most common cause of postinfectious bronchiolitis obliterans, with one study finding that bronchiolitis obliterans developed in 36% of children with adenoviral acute lower respiratory tract infection [12]. Castro-Rodriguez and colleagues found that 47% of children who had adenovirus pneumonia developed evidence of bronchiolitis obliterans on chest computed tomography at five-year follow-up [13].

Adenoviruses have emerged as significant opportunistic pathogens in the immunocompromised population, with infections ranging from asymptomatic to disseminated disease with fatalities [1]. Mortality rates in immunocompromised populations with adenoviral infection are highly variable, ranging from 2% to 69% [14–20], with rates as low as 2% in prospective screening studies where a large portion of the patients are asymptomatic [14]. Conversely, mortality rates approaching 70% have been documented in hematopoetic stem cell transplant (HSCT) patients with symptomatic disease [17, 18]. Prior studies have suggested that adenoviral respiratory infection in the immunocompromised patient may be associated with substantial mortality [15, 16, 21]. We investigated the morbidity and case fatality in immunocompromised children with severe adenoviral respiratory infection as compared to immunocompetent children, as well as the long-term respiratory morbidity in survivors.

## 2. Materials and Methods

We performed a combined retrospective-prospective cohort study of all immunocompromised patients admitted to the intensive care unit (ICU), either pediatric ICU or cardiac ICU, with adenoviral respiratory infection at four tertiary care children's hospitals between August 2009 and October 2013. We also performed a secondary matched case control analysis matching our cohort of immunocompromised patients at a 1 : 1 ratio on age and severity of illness score with immunocompetent patients with adenoviral respiratory infection. Adenoviral respiratory infection was defined as the identification of adenovirus from a nasopharyngeal or endotracheal specimen by polymerase chain reaction (PCR), direct fluorescent antibody, or shell viral culture with signs and symptoms of respiratory illness (e.g., supplemental oxygen requirement, wheezing, and cough) at the time of specimen collection. Immunocompromised state was defined as immune system dysfunction as a result of medications after solid organ transplant (SOT) or HSCT, neutropenia with absolute neutrophil count (ANC) less than 500 cells/mm^3^, congenital immunodeficiency, or human immunodeficiency virus. The institutional review boards at the Children's National Medical Center, Wake Forest Baptist Medical Center, Hackensack University Medical Center, and University of Maryland Medical Center reviewed and approved this study.

Review of clinical, administrative, and laboratory databases was conducted to collect information on patient characteristics and outcomes. Clinical data collected included hospital and ICU length of stay; presence of respiratory failure, multiple organ dysfunction syndrome (MODS), acute respiratory distress syndrome (ARDS), concomitant viral, bacterial, or fungal infection, and other chronic conditions; requirement of mechanical ventilation, renal replacement therapy (RRT), or extracorporeal life support (ECLS); absolute lymphocyte count (ALC) at time of infection; and death before hospital discharge. Case fatality was the primary outcome of interest. Mechanical ventilation use was defined as the initiation of positive pressure ventilation, which includes both invasive modes (e.g., pressure-control ventilation, high frequency oscillatory ventilation) and noninvasive modes (e.g., bilevel positive airway pressure). MODS was established based on the International Pediatric Sepsis Consensus Conference criteria [22]. The Berlin Definition for Acute Respiratory Distress Syndrome was used to establish a diagnosis of ARDS [23]. The Paediatric Index of Mortality 3 (PIM3) was calculated for each patient at the time of admission to the ICU to establish a severity of illness index and corresponding predicted probability of death [24].

We defined viral coinfection as the identification of other viral agents by PCR, direct fluorescent antibody testing, or shell viral culture testing on the same nasopharyngeal, endotracheal, or bronchoalveolar lavage specimen that identified adenovirus. All three testing modalities could detect, in addition to adenovirus, respiratory syncytial virus, influenza A and B, and parainfluenza viruses 1–3. Respiratory viral PCR could additionally detect human metapneumovirus and human rhinoviruses and enteroviruses. We defined bacterial or fungal coinfection as the identification of a bacterial or fungal pathogen in culture from an endotracheal, bronchoalveolar, or sterile blood specimen. Respiratory morbidity was assessed either retrospectively or prospectively at one year following diagnosis of infection. Review of surviving patient's charts was conducted to assess for chronic respiratory diagnoses, requirement of chronic mechanical ventilation, supplemental oxygen requirement, and chronic respiratory medication use. Chronic respiratory diagnosis was defined as any long-term pulmonary diagnosis made by a physician. Requirement of chronic mechanical ventilation or supplemental oxygen was defined as the need for either modality for at least part of every day (e.g., overnight). Chronic respiratory medications were defined as an inhaled medication prescribed to treat or prevent a respiratory disease for at least one month.

In the primary outcomes analysis, continuous variables were compared using Student's *t*-test or Wilcoxon rank sum testing as appropriate and categorical variables were compared using Fisher's Exact testing. In the matched case control analysis, continuous variables were compared using paired *t*-test or Wilcoxon matched pairs signed rank test and categorical variables were compared using McNemar's test. Type-one error was set at 0.05. All calculations were performed using Stata/IC 12.1 (Stata Corporation, College Station, TX).

## 3. Results

There were 19 immunocompromised patients included in our analysis. Seventeen of the patients were diagnosed by respiratory viral PCR, one by direct fluorescent antibody, and one by shell vial culture. Patient characteristics are presented in Table 1 with detailed patient descriptions presented in Table 2. The most common diagnosis was immunocompromised state in the setting of HSCT (47%). The median age of patients was 3.9 years (interquartile range (IQR) 2.6 to 12.7). The median hospital length of stay was 45 days (IQR 17 to 112) and median ICU length of stay was 20 days (IQR 7 to 48). The median PIM3 predicted mortality for the entire cohort was 5.4% (IQR 1.1% to 5.7%).

Seventeen of the immunocompromised patients (89%) required mechanical ventilation with a median duration of ventilation of 12 days (IQR 1–27 days). Seven (37%) patients met diagnostic criteria for ARDS and 11 (58%) for MODS. RRT was required for seven patients (37%) and one patient (5%) underwent ECLS. Eleven patients did not survive to discharge yielding a case fatality rate of 58%. Of the eleven nonsurviving patients, eight were immunocompromised due to HSCT (case fatality 89%), one due to SOT (case fatality 33%), two due to neutropenia (case fatality 67%), and none due to congenital immunodeficiencies.

Seven patients (37%) had identification of at least one additional virus on the same specimen that detected adenovirus. All seven patients had detection of human rhino-/enterovirus. One patient had detection of respiratory syncytial virus in addition to human rhino-/enterovirus. Three patients (16%) had bacterial coinfection and no fungal coinfections were identified.

Antiviral medication was administered to 13 patients (68%). Cidofovir was the most common antiviral medication given, with administration to 10 patients (53%). Two of those patients were changed from cidofovir to the liposomal formulation and one patient was changed to the enteral formulation, brincidofovir. Other antiviral medications given either for prophylaxis or treatment included acyclovir given to five patients (26%), ganciclovir given to three patients (16%), valacyclovir given to two patients (11%), foscarnet given to two patients (11%), and ribavirin given to one patient (5%).

We compared patient characteristics and clinical features between nonsurvivors and survivors (Table 3). Patient characteristics that were associated with mortality were the cause of immunocompromised state (*p* = 0.015). Clinical characteristics associated with mortality included meeting diagnostic criteria for MODS (*p* = 0.001), requiring RRT (*p* = 0.01), PIM3 predicted mortality (*p* = 0.011), and treatment with cidofovir (*p* = 0.005).

We performed a matched case control analysis of immunocompromised cases and immunocompetent controls with severe adenoviral respiratory infection (Table 4). All 19 controls were diagnosed by respiratory viral PCR. There were no differences in age, gender, or PIM3 predicted mortality between cases and controls. There were no differences in hospital or ICU lengths of stay or rates of respiratory failure. As compared to the immunocompetent controls, the immunocompromised cases were more likely to have MODS (*p* = 0.01), require RRT (*p* = 0.02), have lower ALC (*p* = 0.004), and not survive to discharge (*p* = 0.004).

Long-term respiratory morbidity was assessed one year after adenovirus infection in survivors. Of the eight immunocompromised patients who survived to discharge, one died three months later. This left seven patients included in the follow-up assessment. Five patients (71%) had at least one chronic respiratory diagnosis. Four of the patients (57%) were prescribed daily inhaled steroid medication. Oxygen supplementation was required in three patients (43%) and three patients (43%) required chronic mechanical ventilation. Of the 18 surviving control patients, one died two months later from an unrelated illness and six were lost to follow-up, leaving 11 patients for analysis. One year after severe respiratory adenoviral infection, three (27%) of the control patients were on chronic mechanical ventilation, though all three were on mechanical ventilation prior to their adenoviral infection. Six of the control patients (55%) were on daily inhaled respiratory medications, and four (36%) had no respiratory diagnosis nor required any respiratory medications.

## 4. Discussion

This multicenter study found that severe adenoviral respiratory infection in the immunocompromised population has high associated morbidity and case fatality. We found a longer ICU length of stay (20 days, IQR 7 to 48) and length of mechanical ventilation (12 days, IQR 1–27 days) than previously reported for critically ill pediatric HSCT patients (ICU LOS 3 days [25] and mechanical ventilation 6 days [26]). Our reported case fatality rate of 58% is one of the higher rates reported for adenoviral infection in the immunocompromised population, supporting suggestions that have been made in prior studies that respiratory adenoviral infection has higher associated mortality than other forms of adenoviral infection in the immunocompromised population [15, 16, 27]. Comparison of our immunocompromised cohort with a matched control group of immunocompetent patients further highlights the high morbidity and mortality for immunocompromised patients with severe respiratory adenoviral infection. The immunocompromised group had more MODS, received more renal replacement therapy, and had higher case fatality (58% compared to 5%).

We found an association between cause of immunocompromised state and case fatality, with the case fatality for the HSCT patients being very high (89%). Similarly, de Mezerville et al. found higher mortality rates for pediatric HSCT patients with adenoviral infection as compared to SOT patients [16]. It is likely that the worse outcomes for the HSCT patients are related to their increased degree of compromised immune function.

Cidofovir is a broad-spectrum antiviral agent that inhibits DNA polymerase to which all adenovirus serotypes have been shown to be susceptible in vitro. Recently, cidofovir has been increasingly used in an off–label manner in the treatment of adenovirus in the immunocompromised population, particularly the HSCT population. Case reports and studies have documented successful treatment of adenoviral infection in the SOT and HSCT populations [14, 28, 29]. Conversely, our study found an association between treatment with cidofovir and mortality. This association is likely related to the severity of illness; patients who were more ill were more likely to be treated with cidofovir than those who were less ill.

A high number of patients in our study had coinfections (42%), with the majority being other respiratory viral coinfections. Several other studies have found high adenoviral coinfection rates, ranging from 20 to 50% [2, 21, 30]. The detection of additional viruses on respiratory viral PCR is potentially confounding in patients with severe viral respiratory illness as it may reflect active infection of multiple viruses or infection with a single virus and detection of viral shedding from a second virus. Human rhinoviruses and enteroviruses, which were the most common viruses codetected, continue to be shed for up to six weeks following active infection in previously healthy children [30, 31]. We did not observe any association between coinfection and case fatality.

Consistent with the published literature, we found long-term respiratory morbidity to be high after respiratory adenovirus infection. Our findings that 55% of surviving immunocompetent and 57% of surviving immunocompromised patients were using daily inhaled respiratory medications one year after adenoviral infection were lower than the 95% using inhaled corticosteroids five years after infection reported in one study [13]. However, our finding that 27% of surviving immunocompetent and 43% of surviving immunocompromised patients utilized supplemental oxygen at one year is higher than the 5–13% seen at long-term follow-up in previous studies [12, 13]. Additionally, our finding that 27% of surviving immunocompetent and 43% of surviving immunocompromised patients required chronic mechanical ventilator support is novel. These findings suggest that patients with severe respiratory adenoviral infection are at high risk for long-term respiratory morbidity and that being immunocompromised may place patients at even higher risk.

This study was limited by its retrospective design and relatively small number of patients. Additionally, three methods of testing for adenovirus were used with varying sensitivity and specificity for adenovirus and other viruses causing coinfection. Although we found no association between coinfections and case fatality there was a high level of coinfections found in this study. This high coinfection rate, as well as the high morbidity in this critically ill population, makes it difficult to fully assess risk factors and confounding comorbidities. Finally, there was heterogeneity in the population studied with variability in patients based on location and services available. A prospective study with a larger number of centers would be able to better delineate risk factors.

## 5. Conclusions

Morbidity and case fatality associated with severe respiratory adenovirus infection in the immunocompromised population is high. This morbidity and case fatality appears to be higher than that of other sites of adenoviral infection and was particularly high in the HSCT population. Respiratory morbidity in survivors one year following adenoviral respiratory infection was considerable.

## Figures and Tables

**Table 1:** Characteristics of immunocompromised patients admitted to the intensive care unit with adenoviral respiratory infection.

Characteristic	Number (%)
Age group	
<1 year	2 (11)
1–5 years	10 (53)
6–10 years	1 (5)
11–18 years	6 (32)
Female gender	11 (58)
Race/ethnicity	
Caucasian	2 (11)
African-American	10 (53)
Hispanic	5 (26)
Asian	2 (11)
Other	0 (0)
Cause of immunocompromised state	
Solid organ transplant	3 (16)
Hematopoetic stem cell transplant	9 (47)
Human immunodeficiency virus	0 (0)
Neutropenia	3 (16)
Congenital immunodeficiency	4 (21)
Mechanical ventilation	17 (89)
Acute respiratory distress syndrome	7 (37)
Multiple organ dysfunction syndrome	11 (68)
Renal replacement therapy	7 (37)
Extracorporeal life support	1 (5)
Mortality	11 (58)

Values expressed as *n* (%).

**Table 2:** Detailed descriptions of immunocompromised patients in analysis.

Case ID	Age (years)	Gender	PIM3 predicted mortality (percent)	Cause of immunocompromised state	Coinfections	MODS	ARDS	RRT	ECLS	Antiviral therapy	Case fatality
1	3	M	5.334	Hematopoetic stem cell transplant—allogeneic due to SCID, no GVHD; immunomodulators: mycophenolate mofetil, methylprednisolone, and cyclosporine	RSV, HRV/EV, and Alpha hemolytic *Streptococcus* in blood culture	Yes	No	Yes	No	Cidofovir, acyclovir	Yes

2	3	F	7.04	Neutropenia (ANC = 140/mm^3^) due to AML	HRV/EV	Yes	Yes	No	No	None	Yes

3	2	F	15.4	Hematopoetic stem cell transplant—liver, pancreas, and small bowel; immunomodulators: methylprednisolone	*Enterobacter* respiratory infection	Yes	Yes	Yes	Yes	Cidofovir, ganciclovir, investigational medication INV-CMX001 (liposomal cidofovir)	Yes

4	14	F	5.3	Hematopoetic stem cell transplant—allogeneic due to AML, possible GVHD; immunomodulators: mycophenolate mofetil and cyclosporine	None	Yes	Yes	Yes	No	Cidofovir, acyclovir, ganciclovir, and valacyclovir	Yes

5	18	F	5.6	Hematopoetic stem cell transplant—allogeneic due to Pre-B Cell ALL, no GVHD; immunomodulators: cyclosporine and methotrexate	HRV/EV	Yes	Yes	Yes	No	Cidofovir, acyclovir	Yes

6	2	F	5.5	Hematopoetic stem cell transplant—autologous due to relapsed retinoblastoma, no GVHD; immunomodulators: none	None	No	No	No	No	Acyclovir	No

7	2	M	1.3	Neutropenia (ANC 360/mm^3^) due to hemophagocytic lymphohistiocytosis	None	Yes	No	Yes	No	None	Yes

8	3	M	1.2	Neutropenia (ANC 0/mm^3^) due to Langerhans cell histiocytosis	None	No	No	No	No	Foscarnet, ganciclovir	No

9	1	F	0.14	Transient hypogammaglobulinemia of infancy	HRV/EV	No	No	No	No	None	No

10	5	M	1.1	Solid organ transplant—kidney; immunomodulators: prednisone, tacrolimus	HRV/EV	No	No	No	No	Valganciclovir	No

11	12	F	5.3	Hematopoetic stem cell transplant—allogeneic due to severe aplastic anemia, grade IV GVHD; immunomodulators: methylprednisolone, daclizumab, infliximab, and mycophenolate	None	Yes	No	Yes	No	Cidofovir, investigational medication INV-CMX001 (liposomal cidofovir)	Yes

12	4	F	6.5	Hematopoetic stem cell transplant—autologous due to neuroblastoma, no GVHD; immunomodulators: none	None	Yes	Yes	No	No	Cidofovir	Yes

13	11	M	5.4	Hematopoetic stem cell transplant—allogeneic due to AML, GVHD; immunomodulators: mycophenolate mofetil, and prednisolone	None	Yes	Yes	Yes	No	Cidofovir, ribavirin	Yes

14	4	F	5.8	Hematopoetic stem cell transplant—allogeneic due to SCID variant, no GVHD; immunomodulators: cyclosporine and mycophenolate mofetil	None	Yes	No	No	No	Cidofovir, foscarnet, and cytogam	Yes

15	3	F	5.4	Hematopoetic stem cell transplant—allogeneic for Sickle Cell Disease, GVHD grade IV; immunomodulators: Methylprednisolone, Extracorporeal photopheresis, tacrolimus, cyclosporine, infliximab, rituximab, and mycophenolate mofetil	None	No	No	No	No	Cidofovir, brincidofovir, and acyclovir	Yes

16	<1	M	1.1	DiGeorge Syndrome	None	No	No	No	No	None	No

17	13	F	1.1	Solid organ transplant—kidney; immunomodulators: prednisone, cellcept, and rapamune	None	Yes	Yes	No	No	Cidofovir	No

18	17	M	1.2	Wiscott-Aldrich Syndrome	HRV/EV	No	No	No	No	None	No

19	8	M	1	Common variable immune deficiency	HRV/EV, Alpha hemolytic *Streptococcus* from BAL sample	No	No	No	No	None	No

AML: acute myeloid leukemia; ANC: absolute neutrophil count; ALL: acute lymphoblastic leukemia; ARDS: acute respiratory distress syndrome; BAL: bronchoalveolar lavage; ECLS: extracorporeal life support; GVHD: graft-versus-host disease; HRV/EV: human rhino-/enterovirus; MODS: multiple organ dysfunction syndrome; RRT: renal replacement therapy; RSV: respiratory syncytial virus; SCID: severe combined immunodeficiency.

**Table 3:** Patient and clinical characteristics stratified by survival.

Characteristic	Nonsurvivors	Survivors	*p* value
	(*n* = 11)	(*n* = 8)	
Age (years)	3.9 (3.0–12.7)	3.9 (1.8–11.2)	0.56
Female gender, *n* (%)	8 (72%)	3 (38%)	0.18
Cause of immunocompromised state, *n* (%)			**0.015**
Solid organ transplant	1 (13%)	2 (25%)	
Blood and marrow transplant	8 (73%)	1 (13%)	
Human immunodeficiency virus	0	0	
Neutropenia	2 (28%)	1 (13%)	
Congenital immunodeficiency	0	4 (50%)	
Hospital LOS (days)	46 (17–145)	45 (19–88)	0.90
ICU LOS (days)	19 (7–48)	24 (7–46)	0.80
Mechanical ventilation	11 (100%)	6 (75%)	0.16
Ventilator days	14 (5–34)	5 (0–21)	0.17
Acute respiratory distress syndrome	6 (55%)	1 (13%)	0.15
Multiple organ dysfunction syndrome	10 (91%)	1 (13%)	**0.001**
Renal replacement therapy	7 (64%)	0	**0.01**
Extracorporeal life support	1 (13%)	0	0.58
PIM3 predicted mortality (percent)	5.6 (5.3–6.5)	1.1 (1.1–3.3)	**0.011**
Antiviral treatment	9 (82%)	4 (50%)	0.32
Treatment with cidofovir	9 (82%)	1 (13%)	**0.005**
Coinfection	4 (36%)	4 (50%)	0.66
Viral coinfection	4 (36%)	3 (38%)	0.38
Bacterial coinfection	2 (18%)	1 (13%)	1
Absolute lymphocyte count (cells/mm^3^)	480 (40–650)	1150 (459–4070)	0.13

Values expressed as *n* (%). Continuous variables are shown as median (interquartile range). ICU: intensive care unit; LOS: length of stay; PIM3: Paediatric Index of Mortality 3.

**Table 4:** Matched case control analysis of immunocompromised cases with severe adenoviral respiratory infection and immunocompetent controls with severe adenoviral respiratory infection.

Characteristic	Immunocompromised cases (*n* = 19)	Immunocompetent controls (*n* = 19)	*p* value
Age (years)	3.9 (IQR 2.6–12.7)	3.6 (IQR 1–8.5)	0.11
Female sex	11 (58%)	12 (63%)	0.74
PIM3 predicted mortality (percent)	5.3 (IQR 1.1–5.6)	3.3 (IQR 1.1–4.6)	0.13
Hospital LOS (days)	45 (IQR 17–112)	13 (IQR 4–34)	0.06
ICU LOS (days)	20 (IQR 7–48)	6 (IQR 3–23)	0.07
Respiratory failure	17 (89%)	14 (74%)	0.45
ALC (cells/mm^3^)	560 (IQR 50–1520)	2490 (IQR 1840–4070)	**0.004**
Bacterial coinfection	3 (5%)	2 (11%)	1.00
Viral coinfection	7 (37%)	4 (21%)	0.45
MODS	11 (58%)	2 (11%)	**0.01**
ARDS	7 (37%)	2 (11%)	1.00
RRT	7 (37%)	0	**0.02**
ECLS	1 (5%)	0	1.00
Case fatality	11 (58%)	1 (5%)	**0.004**

Values expressed as *n* (%). Continuous variables are shown as median (interquartile range). PIM3: Paediatric Index of Mortality 3; ICU: intensive care unit; LOS: length of stay; ALC: absolute lymphocyte count; MODS: multiple organ dysfunction syndrome; ARDS: acute respiratory distress syndrome; RRT: renal replacement therapy; ECLS: extracorporeal life support.
